# Two Siblings with the Same Severe Form of 21-Hydroxylase Deficiency But Different Growth and Menstrual Cycle Patterns

**DOI:** 10.3389/fped.2017.00035

**Published:** 2017-03-01

**Authors:** Mariarosaria Lang-Muritano, Karine Gerster, Susanna Sluka, Daniel Konrad

**Affiliations:** ^1^Department of Endocrinology and Diabetology, University Children’s Hospital, Zurich, Switzerland; ^2^Children’s Research Centre, University Children’s Hospital, Zurich, Switzerland; ^3^Swiss Newborn Screening Laboratory, University Children’s Hospital, Zurich, Switzerland

**Keywords:** adrenal hyperplasia, congenital, adult height, hydrocortisone treatment, constitutional delay, *in utero* dexamethasone

## Abstract

Congenital adrenal hyperplasia (CAH) is one of the most frequent autosomal recessive diseases in Europe. Treatment is a challenge for pediatric endocrinologists. Important parameters to judge the outcome are adult height and menstrual cycle. We report the follow-up from birth to adulthood of two Caucasian sisters with salt-wasting CAH due to the same mutation, homozygosity c.290-13A>G (I2 splice), in the 21-hydroxylase gene. Their adherence to treatment was excellent. Our objective was to distinguish the effects of treatment with hydrocortisone (HC) and fludrocortisone (FC) on final height (FH) from constitutional factors. The older girl (patient 1), who showed virilized genitalia Prader scale III–IV at birth, reached FH within familial target height at 18 years of age. Menarche occurred at the age of 15. Her menstrual cycles were always irregular. Total pubertal growth was normal (29 cm). She showed a growth pattern consistent with constitutional delay. The younger sister (patient 2) was born without masculinization of the genitalia after her mother was treated with dexamethasone starting in the fourth week of pregnancy. She reached FH at 16 years of age. Her adult height is slightly below familial target height. Menarche occurred at the age of 12.5, followed by regular menses. Total pubertal growth was normal (21 cm). The average dose of HC from birth to FH was 16.7 mg/m^2^ in patient 1 and 16.8 mg/m^2^ in patient 2. They received FC once a day in doses from 0.05 to 0.1 mg. Under such therapy, growth velocity was normal starting from the age of 2.5 years with an overall average of +0.2 SD in patient 1 and −0.1 SD in patient 2, androstenedione levels were always within normal age range. Similarly, BMI and blood pressure were always normal, no acne and no hirsutism ever appeared. In conclusion, two siblings with the same genetic form of 21-hydroxylase deficiency and excellent adherence to medication showed different growth and menstrual cycle patterns, rather related to constitutional factors than to underlying CAH. In addition, the second patient represents an example of successful *in utero* glucocorticoid treatment to prevent virilization of the external genitalia.

Congenital adrenal hyperplasia (CAH) is one of the most frequent autosomal recessive diseases in central Europe. The reported incidence in Switzerland since the introduction of neonatal screening in 1992 is approximately 1:9,500 births.^1^ The reported worldwide incidence is approximately 1:16,000 births (1). Treatment remains a challenge for pediatric endocrinologists. The daily production rate of cortisol is approximately 8 mg/m^2^/day. Because of enterohepatic circulation, a hydrocortisone (HC) replacement dose of at least 10–12 mg/m^2^/day is required. However, a dose of at least 15 mg/m^2^/day is mostly needed to reach ACTH suppression in children with CAH. Recent data support the recommendation that the daily HC dose should not exceed 17 mg/m^2^ to optimize pubertal growth (2). Over the last three decades, a trend toward increased final height (FH) was observed (3), possibly due to a progressive reduction of total daily dose of glucocorticoids used.

In CAH, the onset of puberty and pubertal development is expected to be normal if glucocorticoid (and mineralocorticoid) supplementation is maintained at adequate levels. Menarche and menstrual cycle are very sensitive parameters, which are surely partly dependent on specific hormonal control of CAH, and also show very wide constitutional variability. Of note, polycystic ovary syndrome (PCOS) is more frequent in poorly controlled patients with CAH (3).

Concerning prenatal prevention of masculinization, antenatal treatment with dexamethasone has been recommended in the 1980s and 1990s (4). More recently, a review of such practice has been advocated because of potential adverse maternal–fetal side effects (5, 6). Currently, antenatal dexamethasone treatment is no longer generally recommended but should be carried out in specialized centers following approved protocols (7).

Herein, we describe and analyze retrospectively the growth pattern and pubertal development of two siblings with the same genetic form of 21-hydroxylase deficiency and excellent adherence to glucocorticoid and mineralocorticoid supplementation from birth to adulthood. The aim of this study was to distinguish the effects of glucocorticoid treatment on FH from possible constitutional factors.

## Methods

Both patients were followed up at our clinic, patient 1 from the age of 6 months and patient 2 from birth. The diagnosis of CAH was based on a virilized genitalia in the first child at birth and prenatal genetic diagnosis in the second one. Regular follow-up appointments took place every 3–6 months at our clinic. Adjustment of glucocorticoid dose was made based on auxological data (linear growth), skeletal maturity (as assessed by the Greulich and Pyle method), and determination of adrenal steroids (total 17-ketosteroids in 24-h-urine collection in patient 1 until the age of 3 years and plasma androstenedione concentrations measured at least every 6 months in both patients).

**Figure 1:**
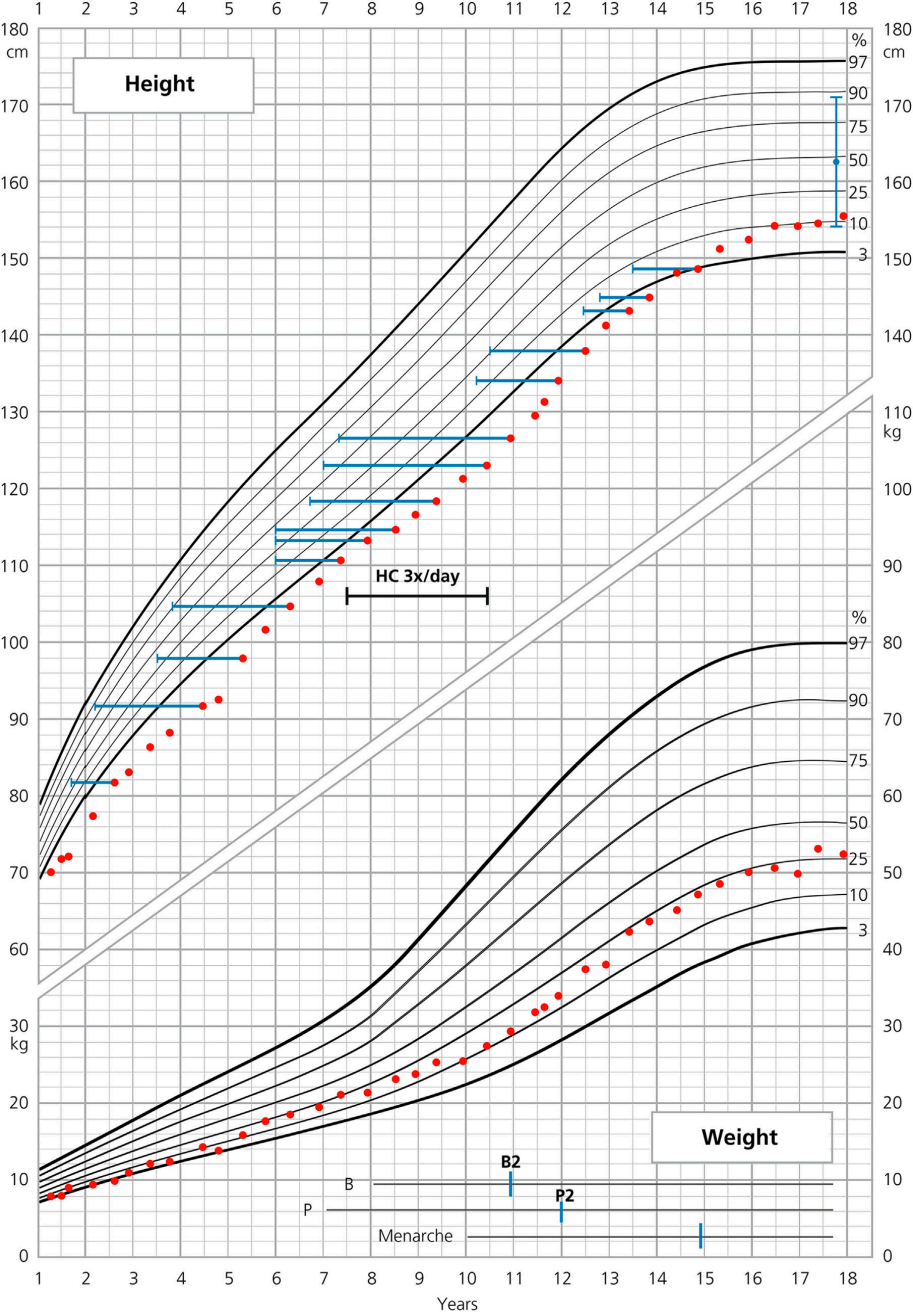
**Growth curve of patient 1**.

**Figure 2:**
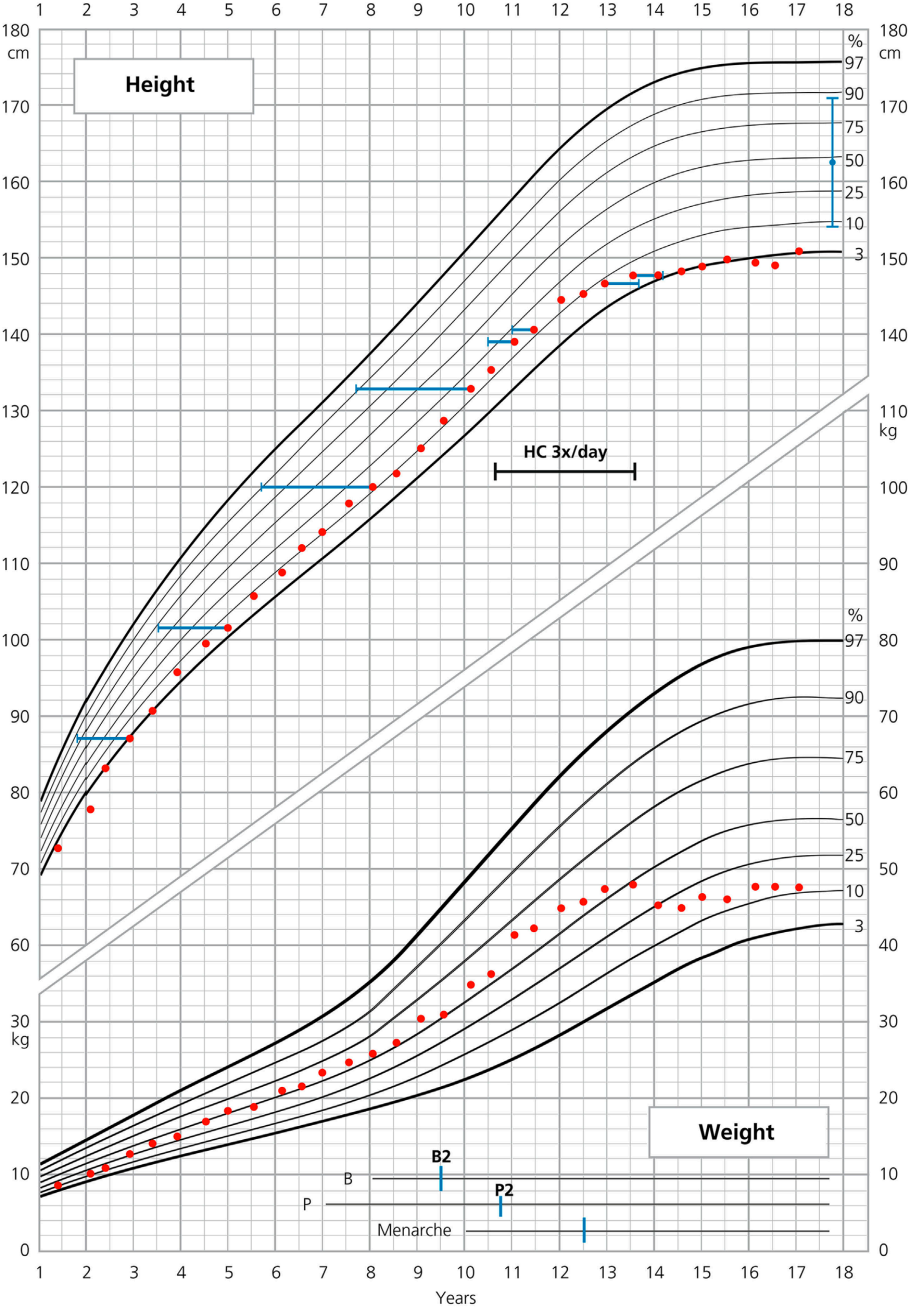
**Growth curve of patient 2**.

Familial target height was calculated as (maternal + paternal height/±13 cm)/2. Total pubertal growth was defined as growth from the breast Tanner stage 2 (B2) until reaching FH. Values of 20.3 ± 6.8 were reported to be normal in females (8). Height and BMI SD scores were calculated with a growth calculator using WHO growth data, and the corresponding growth standard curves are shown in Figures 1 and 2.^2^ The ratio sitting height/subischial leg length × 100 SD was calculated according to Prader et al. (8).

Linear relationships were assessed by Spearman rank correlation analyses. *p* < 0.05 was considered significant.

Androstenedione was measured by Chemiluminescence Immunoassay on an Immulite 1000 immunoanalyzer (Siemens Healthcare GmbH, Erlangen, Germany) with a measuring range of 1.05–35.0 nmol/l and a coefficient of variation of 10.4% at 4.1 nmol/l. Profiling of steroid hormones and urinary steroids was done by gas chromatography mass spectrometry (GC-MS) on a PolarisQ (Finnigan, Brechbühler, Schlieren, Switzerland) supplied with a Restek™ RTX™-1 MS column (Fischer Scientific, Reinach, Switzerland) according to methods reported by Shackleton (PMID:3525596). The urine samples were prepared by pre-extraction of steroids (Chromabond C18ec; Macherey-Nagel AG, Oensingen, Switzerland), enzymatic hydrolysis with β-glucuronidase/arylsulfatase (Roche, Rotkreuz, Switzerland), extraction from the hydrolysis mixture (Chromabond C18ec), derivatization with methoxylamine hydrochloride and trimethylsilylimidazole (Sigma-Aldrich Chemie GmbH, Buchs, Switzerland), and gel filtration (Lipidex 5000; Perkin Elmer AG, Schwerzenbach, Switzerland) before analysis was done by GC-MS. *CYP21A2* mutation analysis was performed using the CAH StripAssay^®^ (ViennaLab Diagnostics, Vienna, Austria) covering the 11 most frequent *CYP21A2* mutations: c.89C>T (P30L), c.290-13C>G (I2 splice), c.329_336del (Del 8bp E3), c.515T>A (I172N), c.[707T>A;710T>A;716T>A] (Cluster E6), c.841G>T (V281L), c.920_921insT (L307 frameshift), c.952C>T (Q318X), c.1066C>T (R356W), c.1357C>T (P453S), and c.1448G>C R483P. *CYP21A2* copy number analysis was performed using the SALSA MLPA P050 CAH probemix (MRC-Holland, Amsterdam, the Netherlands). Reference Sequence NM_000500.6 was used.

Both patients and their parents gave written informed consent to recently re-performed mutation analysis and to the publication of their data.

## Case Reports

### Patient 1

She was born at term with a birth weight of 3210 g (−0.04 SD) and length of 49 cm (−0.07 SD). Pregnancy had been uneventful. She showed virilized genitalia Prader scale III–IV at birth. Karyotype was 46, XX. At the first day of life, plasma cortisol concentration was slightly diminished (187 nmol/l), and ACTH was elevated (74 pmol/l, normal values 2–11.4). Plasma 17-OHP level was elevated at the first (934 nmol/l) and fourth day (54 nmol/l) of life. A diagnosis of CAH was made, and appropriate treatment was begun with HC from the fourth day of life. At day 6 of life, she developed signs of salt-wasting (SW) and fludrocortisone (FC) treatment was added. Under HC, FC, and salt replacement, 17-OHP concentration had diminished to 53 nmol/l and androstenedione to 10 nmol/l at day 25 of life. Molecular analysis revealed a homozygous mutation c.290-13A>G (I2 splice) in the 21-hydroxylase gene. Surgery with correction of the urogenital sinus and clitoroplasty was performed at 6 months of age. During infancy, her weight gain was normal; however, she showed decelerated growth: at 4 months of age, her length was already below the third percentile (−3.2 SD). At the age of two, her height was −2.9 SD, at onset of puberty −2.8 SD. She showed a normal total pubertal growth of 29 cm. She reached an FH of 155.5 cm (−1.1 SD), corresponding to −1 SD below parental target height [height of mother: 165 cm, height of father: 173 cm; resulting familial target height: 162.5 cm (154–171 cm) (−0.1 SD) (Table 1)]. The patient shows rather short legs with SH/SILL × 100 = +2.3 SD.

**Table 1:** **Comparison of given auxological parameters between a published cohort of 38 female patients with salt-wasting (SW)–congenital adrenal hyperplasia (CAH) and our two patients**.

	H-SDS at 2 years	H-SDS at puberty onset	H-SDS at final height (FH)	Total pubertal growth (cm)	_ΔH-SDS_—_FH H-SDS at puberty onset_	_ΔBA_ —_CA at puberty onset_
SW–CAH (38 F) Cohort of Bonfig et al. (2)	−0.4 ± 0.2	0.03 ± 1.4	−0.8 ± 0.9	13.8 ± 7.6	−0.5 ± 0.8	0.2 ± 1.3
Patient 1	−2.9	−2.8	−1.1	29.0	1.6	−3.6
Patient 2	−2.6	−1.0	−1.8	21.0	−0.8	−2.8

Bone age (BA) was retarded during the whole follow-up [between chronological age 2.6 and 14.4 years, average difference between BA and chronological age (CA) (ΔBA–CA) was −1.9 years] (Figure 1). Thelarche (B2) started at the age of 10.9, with a ΔBA–CA of −3.6 years at that time, and pubarche (P2) started at the age of 12. Menarche occurred at the age of 14.9, followed by 1 year of amenorrhea. At the age of 16, polymenorrhea with hypomenorrhea developed. At that time, there were no other clinical (no acne, no hirsutism) or biochemical (no hyperandrogenemia, normal ratio of LH/FSH) signs of PCOS. Initially, progesterone replacement therapy was successful but had to be stopped because of subjective side effects (nausea). Eventually, a contraceptive pill was introduced. Delayed BA fits well to a history of constitutional delay of growth and development of her mother with menarche at 15 years of age. In addition, she reported a tendency to anovulatory cycles over years.

The patient’s average HC supplementation dose was 16.8 mg/m^2^/day from birth to 18 years of age given twice/day or for a limited time three times/day (Figure 1). From 0 to 2 years, her average dose was 20 mg/m^2^/day, from 2 to the onset of puberty at 10.9 years of age 16.3 mg/m^2^/day and during puberty to FH 15.9 mg/m^2^/day. FC was initially 0.075 mg/day, reduced to 0.05 mg/day at 1.5 years of age and augmented to 0.1 mg/day at 4.8 years of age. BMI throughout childhood and adolescence oscillated between +0.1 and +1 SD with a BMI of 21.7 kg/m^2^ (+0.1 SD) at the age of 18. During the entire follow-up period, the patient remained normotensive. As known, overweight and hypertension can be long-term side effects of HC and FC therapy (9, 10).

Starting in infancy, she suffered from at least four well-documented severe adrenal crises of weakness with apathy, vomiting, and hypoglycemia during the initial phase of intercurrent infectious diseases. These episodes were treated by the parents with immediate intramuscular injections of HC. The last crisis occurred at 11.5 years of age.

Laboratory results confirmed excellent adherence of the patient to the treatment regimen. Urinary 17-ketosteroids were only once in the upper normal range at 2 years of age, otherwise always in the low-to-mid normal range. Androstenedione was always normal, mostly <1 nmol/l in prepuberty and thereafter between 1 and 3.8 nmol/l.

### Patient 2

Starting in the fourth week of pregnancy, the mother received dexamethasone 3 × 0.5 mg daily. The diagnosis of classical CAH was confirmed in the fourth month of pregnancy by molecular genetic analysis and, thus, treatment was continued until the end of pregnancy. Otherwise, pregnancy was uneventful and the mother showed no side effects of dexamethasone therapy. The girl was born at term with normal female genitalia. 17-OHP was elevated with a value of 475 nmol/l. She developed signs of SW (Sodium 125 mEq/l, potassium 5.5 mEq/l) at day 6 of life and treatment with HC, FC, and salt was initiated.

Similar to her elder sister, she grew more slowly during the first 6 months of life. At 6 months of age, her length was below the third percentile (−2.6 SD). At 2 years of age, her height was −2.6 SD, at onset of puberty −1 SD. She showed a normal total pubertal growth of 21 cm (Table 1). She reached an FH of 151 cm (−1.8 SD), corresponding to −1.7 SD to target height. Like her elder sister, she also has rather short legs with SH/SILH × 100 = +2.4 SD.

BA was retarded until the age of 12 (between 2.9 and 12 years of age, ΔBA–CA was −1.5 years), thereafter, slightly advanced (between 12 and 13.5 years of age, ΔBA–CA was +0.7 years) (Figure 2). Thelarche appeared at 9.5 years of age, with a ΔBA–CA of −2.5 at that time and pubarche at 10.8 years. Breast development progressed rapidly. Menarche occurred at 12.5 years of age, followed by regular menses.

The patient’s average HC supplementation dose was 16.8 mg/m^2^/day from birth to the age of 17, given twice/day or for a limited time three times/day (Figure 2). From 0 to 2 years, her average dose was 17.9 mg/m^2^/day and during puberty 17.1 kg/m^2^/day. HC dose at the beginning of puberty was 14.3 mg/m^2^/day. FC dose was 0.1 mg/day throughout childhood and adolescence. BMI remained stable throughout childhood and puberty at +1 SD (P75-90) with a BMI of 21 kg/m^2^ (0 SD) at the age of 17. During the entire follow-up period, the patient remained normotensive.

Like her sister, she suffered from adrenal crises with apathy, vomiting, and hypoglycemia, which were treated by her parents with immediate intramuscular injections of HC. During one of these crises at 18 months of age, after a short history of diarrhea and vomiting starting 2 days earlier, she was hospitalized in a soporific but only slightly dehydrated state. Plasma sodium concentration was 125 mmol/l and potassium concentration was 3.8 mmol/l. After administration of HC 50 mg and sodium chloride intravenously, she recovered rapidly and completely. The crisis was interpreted as SW. The last of this kind of crisis occurred at the age of 6.6.

Laboratory results confirmed excellent adherence to treatment since androstenedione concentrations always remained within normal limits, <1 nmol/l during childhood and early puberty and thereafter between 1.3 and 4.9 nmol/l.

## Discussion

The two patients described with an SW form of 21-hydroxylase deficiency attained a normal but rather short adult height. The detailed analysis of the data of the two siblings may suggest that constitutional genetic factors influenced FH more strongly than SW–CAH itself and its related treatment.

Excellent adherence to glucocorticoid and mineralocorticoid treatment, also during puberty, is a very important feature in our two patients and makes them suitable for reliable auxological analysis. The results of such an analysis were partly compared with those published in two large cohorts of females with SW–CAH (2, 11) (Tables 1 and 2). As depicted in Table 1, heights of our patients were lower at 2 years of age and similar or slightly lower at the onset of puberty. Interestingly enough, total pubertal growth gain was higher in our patients. Nevertheless, their FHs were rather in the lower range. As depicted in the corresponding growth curves (Figures 1 and 2), BA was quite delayed during childhood in both patients. Patient 1 showed a normal onset of puberty at the age of 11 and menarche occurred almost 4 years later. However, patient 2 started puberty rather early despite delayed BA and menarche occurred 3 years later. Such different behavior may be due to different degrees of inherited predisposition to constitutional delay.

**Table 2:** **Comparison of given parameters [hydrocortisone (HC)-dose and BMI] between a published cohort of seven female patients with salt-wasting (SW)–congenital adrenal hyperplasia (CAH) and our two patients**.

	HC mg/m^2^/day			BMI SDS		
	0 → 2 years	2 years of age → P2	P2 → final height	0 → 2 years	2 years → P2	P2 → final height (FH)
SW–CAH (13 F) Cohort of Manoli et al. (11)	28.2 ± 7.2	14.7 ± 2.5	16.8 ± 3.6	−0.26 ± 2	1.2 ± 1.1	1 ± 1.1

	**0 → 2 years**	**2 years of age → B2**	**B2 → final height**	**At 2 years**	**At start of puberty**	**At FH**

Patient 1	20.0	16.3	15.9	0.2	0.5	0.14
Patient 2	17.9	16.0	17.1	0.8	1.0	−0.03

On the other hand, it may be argued that the delay in BA during childhood demonstrated in both siblings might be the consequence of glucocorticoid overtreatment.

As partly depicted in Table 2, the average HC doses in our patients were rather in the lower range of HC dose reported in large cohorts in the literature (2, 11, 12). The average HC dose patient 1 received from birth to 2 years, was 20 mg/m^2^, calculated including the dose of approximately 30 mg/m^2^ in the first 2 months of life (given in another center), which was promptly reduced to a mean dose of 16.2 mg/m^2^, given twice a day from the sixth month of life on. The average HC dose in patient 2 from birth to 2 years was 17.9 mg/m^2^, also calculated from birth on. The few specific data for that period of life in the literature show mostly quite more than 20 mg/m^2^/day (11). The very early growth deceleration during the first years of life observed in both patients was followed by completely normal growth velocity during childhood beyond the first 2 years of life (+0.2 SD in patient 1 and −0.1 SD in patient 2), which further supports the notion of constitutional delay.

Of note, a significant correlation was found between HC dose and growth velocity SD during childhood and adolescence (*r*^2^ = 0.27, *p* = 0.034 in patient 1; *r*^2^ = 0.52, *p* = 0.0023 in patient 2). Such correlation may be interpreted differently. Either as a good match between dose adjustment and growth velocity or it may reflect a constant overtreatment, which prevented catch up growth. However, the latter interpretation is not compatible with the good pubertal growth spurt observed in both patients (Table 1) (2).

Other important arguments against a glucocorticoid overtreatment are the low-to-normal blood pressure and the normal BMI observed in both siblings. FC dose could be maintained in our patients at ≤0.1 mg/day, which was surely convenient for blood pressure in the long term (9).

The origin of the slightly altered body proportions in both girls with rather short legs remains unclear. Even though both parents showed normal body proportions, familiarity cannot be excluded since both grandmothers and paternal grandaunts were of rather short stature (150–155 cm). Unfortunately, body proportions of these relatives could not be investigated. However, in the complete absence of any clinical signs of iatrogenic glucocorticoid excess, HC overtreatment seems an unlikely cause for this observation. Nevertheless, selective sensitivity of long bones to the effect of glucocorticoids in the first years of life cannot be definitely excluded.

During childhood, both siblings developed recurrent episodes of SW. The underlying homozygous mutation c.290-13A>G (I2 splice) in the 21-hydroxylase gene was previously described to result either in SW–CAH (78.6%) or in a simple virilizing form (approximately 20%) (13, 14). Apparently, our patient seems to belong to the group with the SW form.

Polycystic ovary syndrome is more common in females with CAH (3). In this regard, the chronically anovulatory cycle pattern observed in patient 1 may suggest PCOS. In fact, no other clinical and laboratory signs for such disease were detected, and adherence to CAH treatment was very good. Given the mother’s history of anovulatory cycles over years, the cycle abnormalities observed in patient 1 may be rather due to familial factors.

In the second sibling, masculinization could be completely prevented by early prenatal dexamethasone treatment of the mother. Nevertheless, the degree of the 21-hydroxylase defect seems to be as severe as in the older sister given the fact that the younger sister developed signs of SW at the sixth day of life and suffered several times from adrenal crises during intercurrent illnesses. No apparent side effects of the prenatal dexamethasone treatment were present neither in the patient nor in the mother, as was previously reported for other cases of prenatal treatment in the literature (15). In particular, the school performance of both siblings is similar and high.

In conclusion, two siblings with the same genetic form of SW-21-hydroxylase deficiency and excellent compliance to glucocorticoid and mineralocorticoid supplementation showed different growth and menstrual cycle patterns, suggesting that constitutional elements strongly influence the outcome, in addition to the disease-specific factors and its related treatment.

## Author Contributions

ML-M treated the patients from birth, made the analysis of the data, and wrote the paper. KG contributed strongly to the analysis of the data. SS is responsible for genetic investigation. DK is the supervisor of the study.

## Conflict of Interest Statement

The authors declare that the research was conducted in the absence of any commercial or financial relationships that could be construed as a potential conflict of interest. The reviewer RG and handling Editor declared their shared affiliation, and the handling Editor states that the process nevertheless met the standards of a fair and objective review.

## Footnotes

^1^www.neoscreening.ch.

^2^www.who.int/childgrowth/standards.
